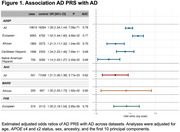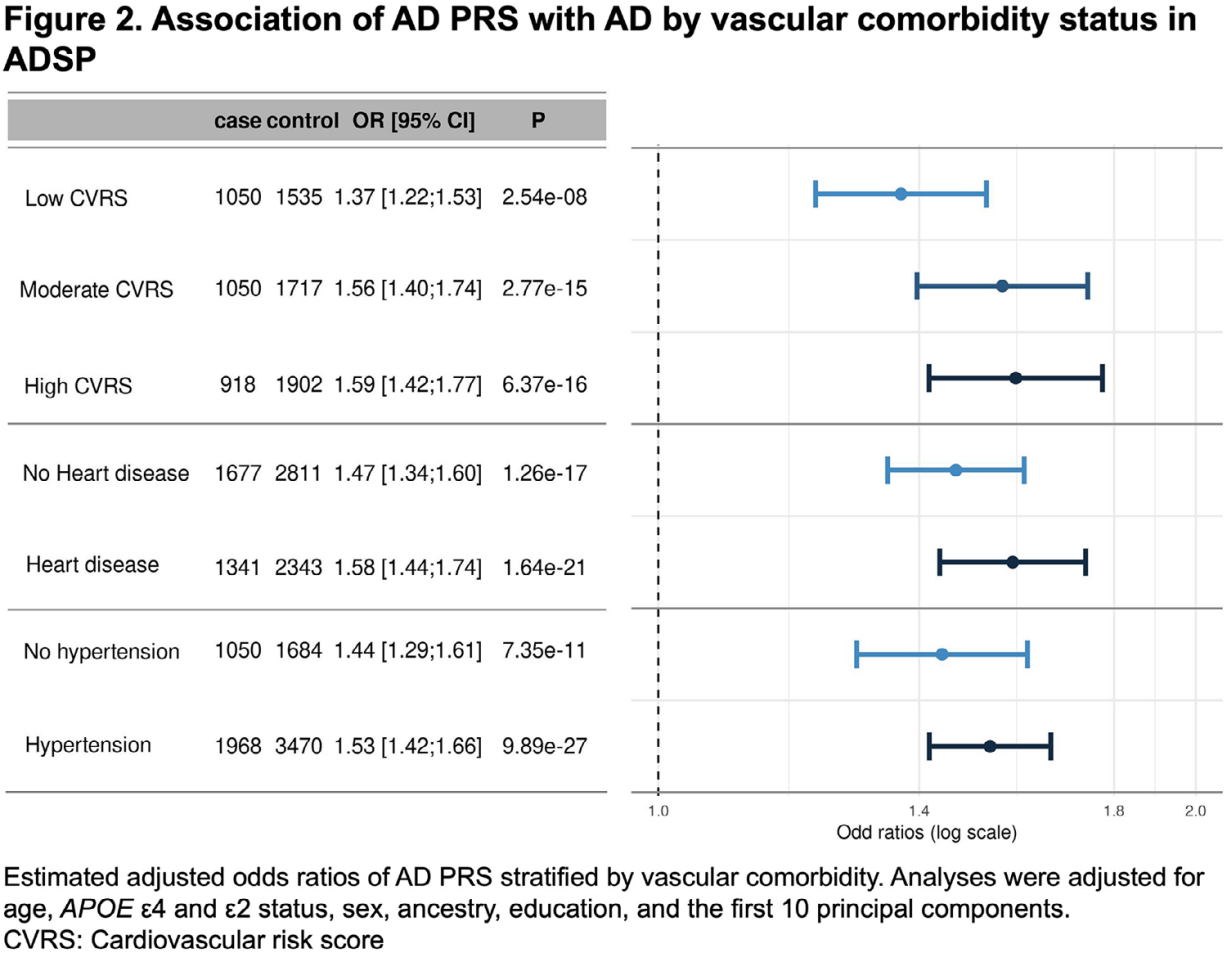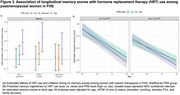# Hormone replacement therapy in women and vascular risk factors modify the association of a multi‐ancestry polygenic risk score with Alzheimer disease and related traits

**DOI:** 10.1002/alz70861_108536

**Published:** 2025-12-23

**Authors:** Nuzulul Kurniansyah, Shinya Tasaki, Congcong Zhu, John J. Farrell, Habbiburr Rehman, Richard Sherva, Richard L. Hauger, Victoria C. Merritt, Matthew S. Panizzon, Rui Zhang, J. Michael Gaziano, Jungsoo Gim, Kun Ho Lee, Ricardo A Vialle, David A. A. Bennett, Lisa L. Barnes, Annie J. Lee, Adam Brickman, Carlos Cruchaga, Paul K Crane, Eden R. Martin, William S Bush, Richard Mayeux, Jonathan L Haines, Margaret Pericak‐Vance, Mark W. Logue, Timothy J. Hohman, Li‐San Wang, Gerald D. Schellenberg, Joanne M Murabito, Ting Fang Alvin Ang, Rhoda Au, Jesse Mez, Kathryn L. Lunetta, Xiaoling Zhang, Lindsay A. Farrer, Alzheimer’s Disease Genetics Consortium, Alzheimer Disease Sequencing Project

**Affiliations:** ^1^ Bioinformatics Program, Boston University, Boston, MA USA; ^2^ Biomedical Genetics, Department of Medicine, Boston University Medical School, Boston, MA USA; ^3^ Rush University Medical Center, Chicago, IL USA; ^4^ National Center for PTSD, VA Boston Healthcare System, Boston, MA USA; ^5^ Center for Behavior Genetics of Aging, University of California, San Diego, La Jolla, CA USA; ^6^ Department of Psychiatry, University of California San Diego, La Jolla, CA USA; ^7^ Center of Excellence for Stress and Mental Health, VA San Diego Healthcare System, San Diego, CA USA; ^8^ Center for Excellence for Stress and Mental Health (CESAMH), VA San Diego Healthcare System, San Diego, CA USA; ^9^ Veterans Affairs San Diego Healthcare System, La Jolla, CA USA; ^10^ University of California San Diego, La Jolla, CA USA; ^11^ Ceneter for Behavior Gentics of Aging, La Jolla, CA USA; ^12^ Division of Aging, Brigham & Women’s Hospital, Harvard Medical School, Boston, MA USA; ^13^ Million Veteran Program (MVP) Coordinating Center, VA Boston Healthcare System, Boston, MA USA; ^14^ Well‐ageing Medicare Institute, Chosun University, Gwangju Korea, Republic of (South); ^15^ Gwangju Alzheimer’s and Related Dementia (GARD) Cohort Research Center, Chosun University, Gwangju Korea, Republic of (South); ^16^ BK21 FOUR, Department of Integrative Biological Sciences, Chosun University, Gwangju Korea, Republic of (South); ^17^ Chosun University, Gwangju, Jeon‐La Korea, Republic of (South); ^18^ Biomedical Science, Chosun University, Gwangju, Gwangju Korea, Republic of (South); ^19^ Korea Brain Research Institute, Daegu Korea, Republic of (South); ^20^ Department of Neurology, Columbia University Medical Center, New York, NY USA; ^21^ Taub Institute for Research on Alzheimer’s Disease and the Aging Brain, Columbia University, New York, NY USA; ^22^ The Gertrude H. Sergievsky Center, College of Physicians and Surgeons, Columbia University, New York, NY USA; ^23^ Department of Neurology, Vagelos College of Physicians and Surgeons, Columbia University, New York, NY USA; ^24^ Washington University in St. Louis, School of Medicine, St. Louis, MO USA; ^25^ NeuroGenomics & Informatics Center, St. Louis, MO USA; ^26^ Department of General Internal Medicine, University of Washington School of Medicine, Seattle, WA USA; ^27^ John P. Hussman Institute for Human Genomics, Miller School of Medicine, University of Miami, Miami, FL USA; ^28^ Department of Population and Quantitative Health Sciences, Institute for Computational Biology, Case Western Reserve University, Cleveland, OH USA; ^29^ The Institute for Genomic Medicine, Columbia University Medical Center, New York, NY USA; ^30^ Department of Psychiatry, Boston University Chobanian & Avedisian School of Medicine, Boston, MA USA; ^31^ Department of Biostatistics, Boston University School of Public Health, Boston, MA USA; ^32^ Vanderbilt Memory and Alzheimer’s Center, Vanderbilt University School of Medicine, Nashville, TN USA; ^33^ Vanderbilt Genetics Institute, Vanderbilt University Medical Center, Nashville, TN USA; ^34^ Department of Pathology and Laboratory Medicine, University of Pennsylvania Perelman School of Medicine, Philadelphia, PA USA; ^35^ Boston University School of Medicine, Boston, MA USA; ^36^ Framingham Heart Study, Boston University Chobanian & Avedisian School of Medicine, Boston, MA USA; ^37^ Slone Epidemiology Center, Boston University Chobanian & Avedisian School of Medicine, Boston, MA USA; ^38^ Department of Anatomy & Neurobiology, Boston University Chobanian & Avedisian School of Medicine, Boston, MA USA; ^39^ Alzheimer’s Disease Research Center, Boston University Chobanian & Avedisian School of Medicine, Boston, MA, USA, Boston, MA USA; ^40^ Department of Epidemiology, Boston University School of Public Health, Boston, MA USA; ^41^ Department of Neurology, Boston University Chobanian & Avedisian School of Medicine, Boston, MA USA; ^42^ Department of Neurology and Ophthalmology, Boston University Chobanian & Avedisian School of Medicine, Boston, MA USA

## Abstract

**Background:**

Alzheimer’s disease (AD) is influenced by genetic and modifiable risk factors, including sex‐specific hormones and vascular comorbidities that may contribute to disparities in disease risk.

**Method:**

We developed an APOE‐independent multi‐ancestry polygenic risk score (PRS) for AD using a PRS‐CS weighted summation approach, integrating summary statistics from European ancestry (EA), African American, and East Asian cohorts from the Alzheimer’s Disease Genetics Consortium. PRS performance was evaluated in the Alzheimer’s Disease Sequencing Project (ADSP) and validated in All of Us, Framingham Heart Study, and Minority Aging Research Study cohorts. We evaluated associations of the PRS with AD risk, cognitive performance and brain MRI measures among individuals with and without several vascular comorbid diseases and with hormone replacement therapy (HRT) use among women who had natural menopause.

**Result:**

The AD PRS was associated with AD in the ADSP sample, with odds ratios per standard deviation ranging from 1.14 to 1.52 across ancestry groups and robust validation in independent cohorts (Figure 1). Stratified analyses showed that the association between PRS and AD risk was stronger among individuals with vascular comorbidities (Figure 2), suggesting that long‐term poor vascular health may amplify polygenic risk for AD. The AD PRS was also associated with memory, executive function, and language performance, and sex‐specific differences in memory score observed among EA individuals. Among individuals with a high PRS (top tertile of the PRS distribution), ever‐users of HRT exhibited better memory trajectories over time compared to never‐users. Timing played a crucial role: HRT initiation within five years of menopause was associated with higher memory scores (β = 0.24, *p* = 3.05×10⁻³; Figure 3A). Trajectory analyses indicated that the association between HRT and memory was most prominent in midlife and attenuated in later years (Figure 3B). AD PRS was also associated with AD‐related brain MRI measures, suggesting its contribution to early structural changes linked to AD.

**Conclusion:**

Our findings demonstrate the utility of an ancestry‐aware PRS for advancing understanding of AD in diverse populations. Future research incorporating AD PRS in studies of sex‐specific, vascular, and hormonal risk factors may offer promise for personalized interventions and prevention strategies for AD